# Controls on pathogen species richness in plants’ introduced and native ranges: roles of residence time, range size and host traits

**DOI:** 10.1111/j.1461-0248.2010.01543.x

**Published:** 2010-12

**Authors:** Charles E Mitchell, Dana Blumenthal, Vojtěch Jarošík, Emily E Puckett, Petr Pyšek

**Affiliations:** 1 Department of Biology, University of North Carolina at Chapel HillChapel Hill, NC 27599-3280, USA; 2 Rangeland Resources Research Unit, USDA-ARS1701 Center Avenue, Fort Collins, CO 80526, USA; 3 Faculty of Science, Department of Ecology, Charles University PragueViničná 7, CZ-128 44 Prague, Czech Republic; 4 Institute of Botany, Academy of Sciences of the Czech RepublicCZ-252 43 Průhonice, Czech Republic

**Keywords:** Biological invasions, CSR plant resource strategy, enemy release hypothesis, introduced species, invasive species, parasite diversity, pathogen species richness, residence time

## Abstract

Introduced species escape many pathogens and other enemies, raising three questions. How quickly do introduced hosts accumulate pathogen species? What factors control pathogen species richness? Are these factors the same in the hosts’ native and introduced ranges? We analysed fungal and viral pathogen species richness on 124 plant species in both their native European range and introduced North American range. Hosts introduced 400 years ago supported six times more pathogens than those introduced 40 years ago. In hosts’ native range, pathogen richness was greater on hosts occurring in more habitat types, with a history of agricultural use and adapted to greater resource supplies. In hosts’ introduced range, pathogen richness was correlated with host geographic range size, agricultural use and time since introduction, but not any measured biological traits. Introduced species have accumulated pathogens at rates that are slow relative to most ecological processes, and contingent on geographic and historic circumstance.

## Introduction

Plant and animal species vary by orders of magnitude in the richness of pathogen and other parasite species known to infect them (Strong & Levin 1975; Cornell & Hawkins 1993; Poulin & Morand 2004; Dobson *et al.* 2008). Much of this variation in parasite richness can be explained by host attributes, including their biological traits, ecological history and geographic distribution (Nunn *et al.* 2003; Poulin & Morand 2004; Ezenwa *et al.* 2006; Lindenfors *et al.* 2007). When hosts are introduced to novel regions, they are generally reported to have lower pathogen richness where they are introduced than where they are native (Mitchell & Power 2003; Colautti *et al.* 2004; Torchin & Mitchell 2004; van Kleunen & Fischer 2009). However, as introduced hosts spread geographically and persist in time, they are expected to accumulate species of pathogens and other natural enemies (Strong & Levin 1975; Cornell & Hawkins 1993; Guegan & Kennedy 1993; Clay 1995; Mitchell & Power 2003; Colautti *et al.* 2004; Poulin & Morand 2004; Torchin & Mitchell 2004; Carroll *et al.* 2005; Mitchell *et al.* 2006; Brändle *et al.* 2008; Perkins *et al.* 2008; van Kleunen & Fischer 2009).

Here, we seek to answer three questions stemming from these observations. First, how long does it take for introduced hosts to accumulate as many pathogen species as they had in their native range? This may be central to the dynamics of biological invasions because enemies accumulated after introduction may impact the outcome of invasions as much as the initial loss of enemies (Mitchell & Power 2003; Colautti *et al.* 2004; Torchin & Mitchell 2004; Carroll *et al.* 2005; Ricklefs 2005; Parker *et al.* 2006; Siemann *et al.* 2006; Thorpe & Callaway 2006; Hawkes 2007; Perkins *et al.* 2008; Carlsson *et al.* 2009; Davis 2009; Kelly *et al.* 2009). Second, what factors control pathogen species richness on introduced hosts? Introduced hosts are notably dynamic in space and time (Strayer *et al.* 2006; Davis 2009; Williamson *et al.* 2009), suggesting that historic and geographic factors may control introduced range pathogen richness. Third, are the factors that control pathogen richness in hosts’ introduced range different from those in the hosts’ native range? Pathogen richness in hosts’ native ranges reflects processes occurring over longer periods of time, which may increase the influence of biological traits. Understanding the factors that control or predict accumulation of pathogens by introduced hosts is important because these pathogens are at high risk of causing emerging infectious diseases of humans, livestock, wildlife and plants (Daszak *et al.* 2000; Anderson *et al.* 2004; Kelly *et al.* 2009). Our results show that for 124 species of plants introduced from Europe to the United States, pathogen species accumulated over centuries. Introduced range pathogen richness was explained by historic and geographic factors, whereas native range pathogen richness was also influenced by host biological traits. These results were not explained by potential confounding factors, including sampling effort and host phylogeny.

## Methods

### Data and predictions

Major biological traits of individual host organisms that have been hypothesized to control parasite richness include body size (Nunn *et al.* 2003; Poulin & Morand 2004; Ezenwa *et al.* 2006; Lindenfors *et al.* 2007), innate resistance to infection (Poulin & Morand 2004) and resource strategy (Blumenthal *et al.* 2009). Larger bodied organisms are hypothesized to support a greater number of parasite individuals, and hence a greater number of species (Poulin & Morand 2004). We quantified body size in terms of plant height, specifically the log-transformed average of the minimum and maximum height at maturity. Data on height were obtained from the BiolFlor database of the German flora (Klotz *et al.* 2002), and from a working database, CzechFlor, of the Czech Flora (Institute of Botany, Průhonice, Czech Republic). We examined one putative resistance trait, a thickened leaf cuticle and epidermis. While ultimately this trait can reflect adaptation to water limitation, proximately it provides physical resistance against infection by many fungal pathogens (Mendgen 1996; Carver & Gurr 2006), particularly powdery mildews (Carver *et al.* 1996). Data on leaf type were obtained from BiolFlor (Klotz *et al.* 2002). Of six canonical leaf anatomies, succulent and scleromorphic leaves are the two types that are defined, in part, by a thickened epidermis and cuticle. We regarded hosts with either succulent or scleromorphic leaf anatomy (including those plants with intermediate leaf types that included either of these anatomies, such as mesomorphic/scleromorphic) as having a thickened epidermis and cuticle relative to the other hosts. Plants adapted to environments with limited soil resources generally have greater constitutive defenses, and thus can also support lower pathogen richness (Blumenthal *et al.* 2009). We used Grime’s evolutionary resource strategy of stress tolerance as an indicator of adaptation to limited soil resources (Grime *et al.* 1997). We regarded plants whose strategy included stress tolerance as being stress tolerant, and plants with other strategies as not being stress tolerant. Data on resource strategy were obtained from the same sources as for height (the BiolFlor and CzechFlor databases). Other traits such as clonal growth and inbreeding may also increase pathogen richness. Pathogen richness is predicted to increase with height, to be decreased by a thickened leaf cuticle and epidermis and decreased by stress tolerance.

The chief historic and geographic attributes of a host that have been hypothesized to control parasite richness include a history of domestication or agricultural use by humans (Clay 1995; Mitchell & Power 2003), the size of its geographic range (Strong & Levin 1975; Clay 1995; Nunn *et al.* 2003; Poulin & Morand 2004; Diez *et al.* 2010), the diversity of habitats in which it occurs (Nunn *et al.* 2003; Poulin & Morand 2004; Ezenwa *et al.* 2006; Lindenfors *et al.* 2007) and the length of time it has been resident in that range, or its residence time (Guegan & Kennedy 1993; Ebert *et al.* 2001; Poulin & Morand 2004; Torchin & Mitchell 2004; Mitchell *et al.* 2006; Thorpe & Callaway 2006; Hawkes 2007; Perkins *et al.* 2008; Diez *et al.* 2010). We obtained data on whether each host had a history of agricultural use from Mitchell & Power (2003; ‘heavily used by humans’, in their terms). We estimated the introduced geographic range size of each host as the sum of the areas of the U.S. states and territories, excluding Alaska due to its disproportionate size, in which the plant was reported to occur (USDA 2008). We similarly estimated the native geographic range size of each host as the sum of the areas of the regions of the Flora Europaea in which the plant was reported to occur (Tutin *et al.* 1964–1980). In the native range of each host, the number of habitat types occupied (habitat richness) is the sum of the number of habitat types (maximum possible = 88) that the host was reported to occupy (based on data from > 24 000 vegetation plots) in the Czech Republic (Sádlo *et al.* 2007). In the introduced range, we obtained data on minimum residence time by searching both primary and secondary sources (Appendix S1) for each hosts’ year of introduction to North America. When year of introduction was not directly estimated, we used the year of first report. When a source listed a range of years as the introduction date, we took the midpoint. For each host, the estimated year of introduction was subtracted from 2003 to yield minimum residence time in the introduced range. Data on host residence time are not relevant to the native range, and data on host habitat richness were not available in the introduced range. Thus, our models of native range pathogen richness included habitat richness but not residence time, and *vice versa* in the introduced range. For this reason, we focused on biological traits when comparing the importance of host attributes between ranges. Increases in each of the four historic/geographic factors are predicted to increase pathogen richness.

We compared the relative importance of these biological and historic/geographic factors in statistically explaining pathogen species richness within each range. We analysed data on fungal and viral pathogens recorded on 124 host plant species native to Europe and naturalized to the United States from Mitchell & Power (2003). They randomly selected 473 hosts from all angiosperm species naturalized (surviving in wild populations) to the United States (introduced range) from Europe (native range). Chiefly using online databases (http://nt.ars-grin.gov/fungaldatabases/fungushost/fungushost.cfm; http://pvo.bio-mirror.cn/refs.htm), they enumerated the rust, smut and powdery mildew fungus species, as well as the virus species, reported to naturally infect each host in each range. These fungi are biotrophic (largely obligate) pathogens that infect leaves, stems and flowers. The 124 hosts analysed here are all those for which we could compile the additional data required to test our hypotheses. In all analyses, the unit of replication was a plant species. While this broad comparative approach provides limited mechanistic insight into any one host, we adopted it to maximize generality.

### Statistical approach

We analysed three response variables: native range pathogen richness, introduced range pathogen richness and proportional release from pathogens in the introduced range (native range richness minus introduced range richness, divided by native range richness). Proportional pathogen release was modelled using grouped binary models that assumed binomial errors and a logit link function. These models assumed that each host had a fixed number of pathogens from which it could be released (its native range pathogen richness). While absolute pathogen release is appropriate for testing predictions based on pathogen pressure in the native range (Blumenthal *et al.* 2009), here we used proportional release to test predictions independent of pathogen pressure in the native range. Models of pathogen richness assumed Poisson errors and a log link function. All generalized linear models were fit using sas/insight 9.1.3 (Cary, NC, USA).

In non-experimental studies, the appropriate statistical model is typically not known *a priori*. Therefore, we used a multimodel statistical approach based on information theory (Burnham & Anderson 2002). We fit three parallel sets of models, one for each of our three response variables. For each response variable, we first fit a global model that included all three biological and all three historic/geographic variables. We did not hypothesize any strong interactions, and searches for unhypothesized patterns in observational datasets are prone to detect spurious correlations, hence we did not include any interaction terms. The global model also controlled for sampling effort, a chief factor that confounds analyses of pathogen richness. Sampling effort was estimated by the number of citations of each host in each range, a standard method in studies of parasite species richness (Nunn *et al.* 2003; Poulin & Morand 2004; Ezenwa *et al.* 2006; Lindenfors *et al.* 2007; Blumenthal *et al.* 2009). More specifically, our method duplicated Blumenthal *et al.* (2009), then we log-transformed the count (+1).

We then fit 14 models that were each a subset of the global model. All subset models also included sampling effort. To assess the importance of biological variables as a group vs. historic/geographic variables as a group, we analysed the two models including all the variables in one of these two categories, and none in the other. To assess the importance of each variable individually, we analysed the six models derived from the global model by individually removing each of the six variables. To assess the importance of biological and historic/geographic variables acting in concert, we examined the three models including each individual historic/geographic variable and the full suite of biological variables, and the three models including each biological variable and the full suite of historic/geographic variables. Including the global model, this provided us 15 models with which to test our hypotheses.

For each model, we calculated Akaike’s information criterion (AIC). Specifically, we calculated the small-sample quasi-likelihood information criterion (QAIC_c_) by hand, adding 2 (for estimation of its intercept, and of 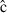 from the global model) to the degrees of freedom to yield the value of *K*. In our analyses, the AIC value of the best model was not sufficiently less than other models to reject all of them. Therefore, we base our results on multimodel inference as well as model selection. Specifically, for each response variable, we calculated the Akaike weight (*w*_*i*_) for each model. Then, for each explanatory variable, we summed the Akaike weights of the models that included that variable. All explanatory variables appeared in an equal number of models. Therefore, the summed Akaike weight of each explanatory variable *j*, *w*_+_( *j* ), indicates its relative importance (Burnham & Anderson 2002).

### Model checking and controls

We checked the fit of each global model. We examined predictive power based on the correlation between the observed and predicted values (native range richness: *R* = 0.70; introduced range richness: *R* = 0.81; proportional release: *R* = 0.45). More formally, we tested the null hypothesis that the model holds by binning observations based on predicted values, and applying a chi-square test. Each test detected some lack-of-fit (native range richness: χ^2^ = 3.98, *P*= 0.046; introduced range richness: χ^2^ = 5.47, *P*= 0.019; proportional release: χ^2^ = 2.09, *P*= 0.15). In Poisson and logistic regression, quasi-likelihood can correct for lack-of-fit resulting from overdispersion of the data. Overdispersion is identified when there is lack-of-fit but the variance inflation factor 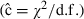 is < 4 (Burnham & Anderson 2002). Each model’s 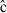 was < 4 (range: 1.80–2.48). Together, these checks indicated that our statistical analyses provided a solid basis for inference.

Geographic range size can be confounded with latitude, which can influence pathogen richness (Clay 1995). To test this, we estimated the mean latitude of each host’s native and introduced geographic ranges. This was calculated as a weighted mean of the central latitude of each U.S. state (or each region of the Flora Europaea) in which the plant was reported to occur, where the weight of each state or region was the difference between its maximum and minimum latitude.

Host phylogeny commonly confounds comparative analyses (Nunn *et al.* 2003; Poulin & Morand 2004), but for many other data sets, it has no detectable influence on parasite richness (Poulin & Morand 2004; Ezenwa *et al.* 2006; Lindenfors *et al.* 2007). We evaluated the influence of host phylogeny by testing the hypothesis that closely related hosts are more similar to one another than expected by chance, with respect to each variable in our data (Nunn *et al.* 2003; Poulin & Morand 2004; Ezenwa *et al.* 2006; Lindenfors *et al.* 2007). To do this, we first used the online program Phylomatic (Webb & Donoghue 2005) to generate a tree based on the phylogeny of Stevens (2001 onwards) with branch lengths estimated from Wikstrom *et al.* (2001). We then adjusted fine-scale topology based on additional published molecular phylogenies (Appendix S2), using Mesquite v.2.6 (Maddison & Maddison 2009). The tree was ultrametricized, and nodes not dated by Wikstrom *et al.* (2001) were adjusted to be distributed evenly between their dates, or between their latest date and the present. Polytomies were resolved to yield zero-length branches. For the resulting finished tree (Figure S1 in Supporting Information), we used the phylosignal function of the ‘picante’ package (v.0.6) in R (v.2.8.1; R Foundation for Statistical Computing, Vienna, Austria) to estimate Blomberg’s *K* statistic (Blomberg *et al.* 2003) in each range. This procedure conducts phylogenetically independent contrasts and compares the observed variance to the variance expected under Brownian evolution, based on 999 random shuffles of the tips of the tree. The *K* statistic is bounded between 0 and 1, where a greater value of *K* (and lesser associated *P*-value) indicates a stronger phylogenetic signal. We corrected for multiple comparisons using the sequential Bonferroni procedure, based on the standard alpha = 0.05.

## Results

### Phylogenetic signal

There was no detectable phylogenetic signal in either native range (*K*=0.146; *z* = −0.338; *P*= 0.41) or introduced range (*K*=0.142; *z* = −0.233; *P*= 0.46) pathogen richness. Among the 13 explanatory variables, there was significant phylogenetic signal for plant height (*K* = 0.353; *z* = −2.87; *P*< 0.001). There was no significant phylogenetic signal for any other explanatory variable (estimates of *K* ranged from 0.15 to 0.25, all much less than for height). Overall, these analyses suggest that host phylogeny had little influence on our results. While many studies of pathogen richness have found strong dependence on host phylogeny (Nunn *et al.* 2003; Poulin & Morand 2004), others have not (Poulin & Morand 2004; Ezenwa *et al.* 2006; Lindenfors *et al.* 2007).

### Pathogen richness in native range

**Figure 1:**
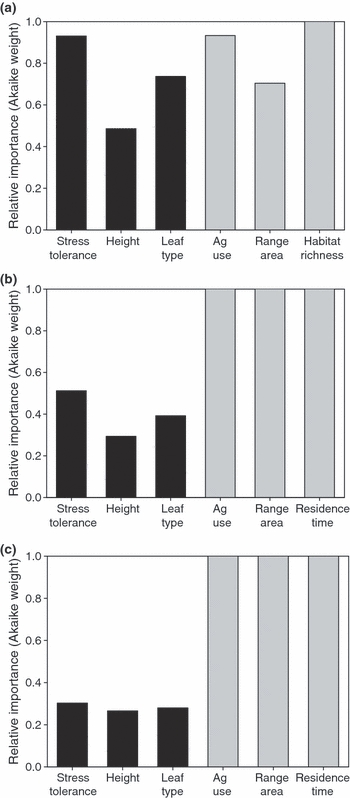
The relative importance of biological (black bars) and historic/geographic (grey bars) variables in explaining (a) pathogen species richness in hosts’ native range, (b) pathogen richness in hosts’ introduced range, (c) proportional pathogen release in hosts’ introduced range (i.e. native range richness minus introduced range richness, then divided by native range richness). Each bar indicates the sum of the Akaike weights of the 11 models that included each explanatory variable. The possible range is from 0 (minimal importance) to 1 (maximal importance). In plants’ native ranges, biological, historic and geographic factors were all important in explaining pathogen richness. In plants’ introduced ranges, pathogen richness and pathogen release were both explained chiefly by historic and geographic, not biological, factors.

In hosts’ native range, pathogen richness was correlated with both biological and historic/geographic factors. The relative importance of the six factors we hypothesized to control pathogen richness is gauged by their Akaike weights summed across the full set of 15 models (Table 1). The summed Akaike weights for host stress tolerance, history of agricultural use and habitat richness were all close to the maximum possible value of 1.0 (weights > 0.93; Fig. 1a). This indicates that these three variables were the most important factors explaining pathogen richness in hosts’ native range. While their summed Akaike weights indicated that host height, leaf type (thickened cuticle and epidermis) and geographic range size were substantially less important in explaining native range pathogen richness, no variable was negligible (weights between 0.48 and 0.74; Fig. 1a). The effect of leaf type was opposite to that hypothesized, hence it does not represent any form of innate resistance within the context of this study.

**Table 1:** Model selection statistics for pathogen species richness in the hosts’ native range. All models also included sampling effort (log-transformed citation count) as an explanatory variable

Biological explanatory variables	Historic and geographic explanatory variables	*K*	Log-likelihood	QAIC_c_	ΔQAIC_c_	Akaike weight
Stress, leaf type	Use, area, habitat richness	8	−136.1	134.2	0	0.291
Stress, height, leaf type	Use, habitat richness	8	−136.5	134.5	0.325	0.247
Stress	Use, area, habitat richness	7	−140.0	135.3	1.06	0.171
Stress, height, leaf type	Use, area, habitat richness	9	−135.9	136.4	2.21	0.097
Stress, height	Use, area, habitat richness	8	−139.8	137.4	3.22	0.058
Stress, height, leaf type	Habitat richness	7	−142.9	137.8	3.59	0.048
Leaf type	Use, area, habitat richness	7	−144.3	139.0	4.80	0.026
–	Use, area, habitat richness	6	−147.0	139.1	4.85	0.026
Stress, height, leaf type	Area, habitat richness	8	−142.5	139.7	5.50	0.019
Height, leaf type	Use, area, habitat richness	8	−144.3	141.2	7.03	0.009
Height	Use, area, habitat richness	7	−147.0	141.3	7.06	0.009
Stress, height, leaf type	Use, area	8	−156.0	151.4	17.1	<0.001
Stress, height, leaf type	Area	7	−165.9	157.5	23.3	<0.001
Stress, height, leaf type	Use	7	−166.2	157.8	23.6	<0.001
Stress, height, leaf type	–	6	−177.1	164.9	30.7	<0.001

QAIC_c_, quasi-likelihood information criterion.

**Figure 2:**
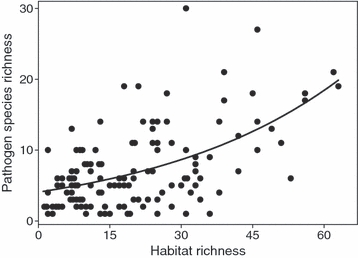
In hosts’ native range, pathogen species richness was greater on hosts that occupied a greater number of habitat types (quasi-likelihood Wald test scaled by the model’s residual deviance divided by its degrees of freedom: χ^2^_1_ = 53.3, *P*< 0.0001). When points had identical *x* and *y* coordinates, the *x*-coordinate was jittered to render all points visible. For simplicity, results from a one-way model are shown. Results were similar in all models analysed, regardless of additional explanatory variables included (e.g. Figure S2).

Pathogen richness in hosts’ native range increased with the number of habitat types occupied by the host. Hosts occupying the greatest observed number of habitat types (63 habitats) were predicted to support over four times as many pathogens as those restricted to a single habitat type (Fig. 2; Figure S2). Also, pathogen richness was, on average, over twice as great on hosts with a history of agricultural use (χ^2^_1_ = 29.8, *P*< 0.0001), and 57% greater on hosts that were not stress tolerant (χ^2^_1_ = 8.61, *P*< 0.0033). In each of the five models that included these three factors, all three had a statistically clear effect (χ^2^_1_ > 5.0, *P*< 0.025) on pathogen richness (e.g. Table S1). These results suggest that host stress tolerance, agricultural use and habitat richness all had largely independent effects, and that this allowed them to jointly explain pathogen richness in the hosts’ native range.

### Pathogen richness and proportional release in introduced range

In the hosts’ introduced range, pathogen richness and proportional release of hosts from pathogens were both explained chiefly by historic/geographic factors, and not biological factors. Summed across all 15 models of pathogen richness (Table 2), the Akaike weights for host stress tolerance, height and leaf type – the three biological factors – were between 0.29 and 0.52. Summed across all 15 models of proportional release from pathogens (Table 3), the Akaike weights for the three biological variables ranged from 0.26 to 0.31. While stress tolerance did not strongly influence proportional pathogen release, it does strongly influence absolute pathogen release (Blumenthal *et al.* 2009), because absolute release is largely determined by pathogen richness in the native range. For both introduced range pathogen richness and proportional release from pathogens, the summed Akaike weights for host history of agricultural use, geographic range size and minimum residence time – the three historic/geographic factors – were all close to the maximum possible value of 1.0 (weights > 0.999). Thus, for both richness and proportional release, the weights for the biological factors were all less than half the weights of the historic/geographic factors (Fig. 1b,c). This indicates that the historic/geographic factors were much more important than the biological factors in explaining both introduced range pathogen richness, and proportional release from pathogens.

**Table 3:** Model selection statistics for proportional pathogen release (native range richness minus introduced range richness, then divided by native range richness) in hosts’ introduced ranges. All models also included sampling effort (difference in log-transformed citation count) as an explanatory variable

Biological explanatory variables	Historic and geographic explanatory variables	*K*	Log-likelihood	QAIC_c_	ΔQAIC_c_	Akaike weight
–	Use, area, time	6	−122.2	111.1	0	0.373
Stress	Use, area, time	7	−121.5	112.8	1.73	0.157
Leaf type	Use, area, time	7	−121.8	113.0	1.93	0.142
Height	Use, area, time	7	−122.0	113.2	2.09	0.131
Stress, leaf type	Use, area, time	8	−121.0	114.7	3.60	0.062
Stress, height	Use, area, time	8	−121.2	114.8	3.70	0.059
Height, leaf type	Use, area, time	8	−121.5	115.1	3.98	0.051
Stress, height, leaf type	Use, area, time	9	−120.4	116.5	5.45	0.024
Stress, height, leaf type	Use, area	8	−135.4	126.2	15.2	<0.001
Stress, height, leaf type	Use, time	8	−136.0	126.7	15.6	<0.001
Stress, height, leaf type	Area, time	8	−136.3	126.9	15.9	<0.001
Stress, height, leaf type	Area	7	−147.3	133.5	22.5	<0.001
Stress, height, leaf type	Use	7	−153.2	138.3	27.2	<0.001
Stress, height, leaf type	Time	7	−176.8	157.3	46.2	<0.001
Stress, height, leaf type	–	6	−189.6	165.3	54.2	<0.001

QAIC_c_, quasi-likelihood information criterion.

**Table 2:** Model selection statistics for pathogen richness in the hosts’ introduced range. All models also included sampling effort (log-transformed citation count) as an explanatory variable

Biological explanatory variables	Historic and geographic explanatory variables	*K*	Log-likelihood	QAIC_c_	ΔQAIC_c_	Akaike weight
–	Use, area, time	6	−99.88	123.6	0	0.217
Stress	Use, area, time	7	−97.86	123.6	0.005	0.216
Leaf type	Use, area, time	7	−98.64	124.4	0.873	0.140
Stress, leaf type	Use, area, time	8	−96.66	124.5	0.955	0.134
Stress, height	Use, area, time	8	−97.25	125.2	1.61	0.097
Height	Use, area, time	7	−99.68	125.6	2.03	0.079
Stress, height, leaf type	Use, area, time	9	−95.87	126.0	2.41	0.065
Height, leaf type	Use, area, time	8	−98.34	126.4	2.82	0.053
Stress, height, leaf type	Use, area	8	−112.1	141.7	18.1	<0.001
Stress, height, leaf type	Use, time	8	−114.2	144.0	20.4	<0.001
Stress, height, leaf type	Area, time	8	−120.4	150.9	27.3	<0.001
Stress, height, leaf type	Use	7	−124.8	153.4	29.9	<0.001
Stress, height, leaf type	Area	7	−127.0	156.0	32.4	<0.001
Stress, height, leaf type	Time	7	−139.4	169.7	46.1	<0.001
Stress, height, leaf type	–	6	−143.1	171.6	48.0	<0.001

QAIC_c_, quasi-likelihood information criterion.

**Figure 3:**
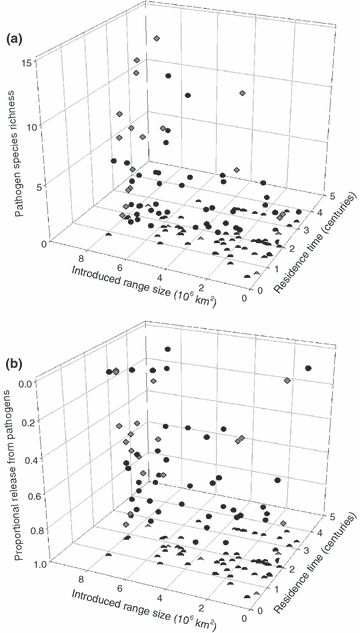
Historic/geographic factors explaining pathogen species richness and proportional release from pathogens in hosts’ introduced range. Black circles indicate hosts with no history of agricultural use, and grey diamonds indicate hosts with a history of agricultural use. Symbols that are only half-visible are located on the *x*–*y* plane (i.e. pathogen richness equals zero or release from pathogens equals one). Chi-square values are from quasi-likelihood Wald tests scaled by the model’s residual deviance divided by its degrees of freedom. Statistics are from the AIC best model for each response variable, which included only sampling effort (log-transformed citation count) and the three explanatory variables shown. Results were similar in all models analysed. (a) Pathogen richness was greater on hosts that had a larger introduced geographic range size (χ^2^_1_ = 34.1, *P*< 0.0001), a longer residence time in the introduced range (χ^2^_1_ = 19.8, *P*< 0.0001) and a history of agricultural use (χ^2^_1_ = 26.6, *P*< 0.0001). (b) Release from pathogens was lesser on hosts that had a larger introduced geographic range size (χ^2^_1_ = 23.8, *P*< 0.0001), a longer residence time in the introduced range (χ^2^_1_ = 15.3, *P*< 0.0001) and a history of agricultural use (χ^2^_1_ = 14.7, *P*= 0.0001). To render all data points visible, the z-axis for pathogen release is reversed, so that 0% release is at the top, and 100% is at the bottom.

Pathogen richness in hosts’ introduced range increased with the host’s introduced geographic range size, and proportional release from pathogens decreased with range size. Hosts with the largest geographic ranges were predicted to have 21 times as many pathogens as those with the smallest geographic ranges (Fig. 3a; Figure S3a), and to be only about half as released from pathogens as those with the smallest ranges (Fig. 3b; Figure S3c). Similarly, pathogen richness increased with hosts’ residence time in the introduced geographic range, and proportional release from pathogens decreased with residence time. The longest established hosts were predicted to host nearly six times as many pathogens as the most recently introduced hosts (Fig. 3a; Figure S3b), and to be only about one-third as released from pathogens as those most recently introduced (Fig. 3b; Figure S3d). To examine whether pathogen richness approached an asymptote with greater residence time, we compared the fit of a model including both a linear and quadratic term for residence time to a model with only the linear term. Adding the quadratic term did not improve model fit (χ^2^_1_ = 0.342, *P*= 0.56). Also, on average, pathogen richness was over five times greater (χ^2^_1_ = 55.3, *P*< 0.0001), and proportional release from pathogens was about one-third less (χ^2^_1_ = 26.7, *P*< 0.0001), on hosts with a history of agricultural use. While geographic range size commonly increases with residence time (Williamson *et al.* 2009), here residence time was not correlated with either geographic range size (Pearson *r* = 0.003, *P*= 0.98) or agricultural use (*t*_122_ = 0.446, *P*= 0.65). However, the geographic range sizes of hosts with a history of agricultural use were, on average, 47% larger than those without (*t*_122_ = −2.92, *P*= 0.0063). Despite this correlation, in each model including all three historic/geographic factors, all three had a statistically clear effect (χ^2^_1_ > 17.0, *P*< 0.0001) on pathogen richness (e.g. Table S2), and a statistically clear effect (χ^2^_1_ > 13.2, *P*< 0.0003) on proportional release from pathogens (e.g. Table S3). These results suggest that agricultural history, range size and residence time all had largely independent effects, and that this allowed them to jointly explain both pathogen richness and proportional release from pathogens in hosts’ introduced range.

### Robustness of results

Because these main results were based on non-experimental analyses, we examined the robustness of the results in detail. While our main results are based on multimodel inference (Fig. 1), identical conclusions are supported by using AIC to select the single best model in each set (Tables 1–3). Identical conclusions are also supported by the traditional approach of comparing the *P*-values in the full model for each response variable to a significance level of *P*= 0.05 (Tables S1–S3). To test whether limiting each analysis to hosts for which data were available for all other analyses biased the results, we re-ran each analysis without this constraint. This yielded similar results (Figure S4). To test whether the difference in the importance of biological factors between the native and introduced range resulted from the difference in the explanatory variables analysed (due to the availability of host habitat richness and residence time data in one range only), we again re-ran our introduced range analyses, but substituting host habitat richness for residence time as an explanatory variable. This also yielded similar results (Figure S5). We tested whether effects of geographic range size on pathogen richness could be explained by latitude. In neither the native (χ^2^_1_ = 1.69, *P*= 0.19) nor the introduced (χ^2^_1_ = 1.23, *P*= 0.26) range did adding latitude to a one-way model of range size increase model fit. We controlled for two chief factors that confound analyses of pathogen richness. First, all of our analyses controlled for sampling effort. This substantially improved model fit, but did not qualitatively alter the results. Second, we tested for effects of host phylogenetic relationships on pathogen richness, and found none. In summary, while our analyses remain correlative, the results were robust to multiple confounding factors, sources of potential bias and methods of analysis.

## Discussion

These results suggest a hypothetical framework for the long-term and large-scale dynamics of pathogen species richness on introduced hosts. Introduced populations are typically founded with a small number of propagules, in one or a few locations, at a given point in time, making co-introduction or subsequent introduction of pathogens a largely stochastic process (Torchin & Mitchell 2004). Introduced populations are also exposed to successive contacts with native species and their pathogens. Both of these processes potentially result in pathogen accumulation. While rates of pathogen introductions and host shifts may be low (Parker & Gilbert 2004), the cumulative probability of pathogen accumulation increases over long periods of time. Thus, recently introduced hosts support uniformly low pathogen richness, whereas longer established hosts may support either low or high pathogen richness. In result, longer established hosts support a greater average number of pathogen species, with the longest established hosts here predicted to support six times as many pathogen species as recently introduced hosts. As introduced populations persist in time, they have the potential to spread geographically (Williamson *et al.* 2009). As they spread, introduced species contact a greater number of other host species, abiotic conditions and habitat types, each combination of which may support different pathogen species, and thus potentially increase pathogen accumulation. The cumulative probability of pathogen accumulation therefore also increases over large regions. As in their native range, introduced populations used in agriculture are planted at higher densities over larger spatial and temporal scales, and thus will be exposed to, and able to support, more species of pathogens. Statistically, host residence time, range size and agricultural use independently influenced pathogen richness (Table S2), yet they act through a common currency: contact between host and pathogen species. Together, these historic/geographic factors were much more important as controls on introduced range pathogen richness than were the biological factors, which can only directly influence infection by pathogens when contact between host and pathogen species has already occurred.

It is recognized that over a long time scale, introduced species integrate into native communities (Strong & Levin 1975; Cornell & Hawkins 1993; Guegan & Kennedy 1993; Torchin & Mitchell 2004; Carroll *et al.* 2005; Mitchell *et al.* 2006; Parker *et al.* 2006; Siemann *et al.* 2006; Strayer *et al.* 2006; Hawkes 2007; Perkins *et al.* 2008; Davis 2009). However, a key question about this process has remained unanswered: How long is the time scale for integration? As a step towards this broad question, we ask: how durable is enemy release? More specifically, how long will it take introduced hosts to recover the same pathogen richness as in their native range? Pathogen richness in the hosts’ native range has accumulated over at least 8000 years, 20 times longer than the maximum in the introduced range, 403 years. These longest established hosts still had 60% fewer reported pathogens in their introduced range than in their native range. Additionally, pathogen richness showed no sign of reaching an asymptote with residence time. Considering these data with the small number of other chronosequences of parasite species richness both shorter (Cornell & Hawkins 1993; Ebert *et al.* 2001; Torchin & Mitchell 2004) and longer (Strong & Levin 1975; Guegan & Kennedy 1993) than ours suggests that parasite richness of introduced hosts may saturate on a roughly millennial scale. Our results support the idea that enemy release does have a limited duration, with its potential benefit decaying over ecological time (Cornell & Hawkins 1993; Guegan & Kennedy 1993; Torchin & Mitchell 2004; Carroll *et al.* 2005; Mitchell *et al.* 2006; Siemann *et al.* 2006; Hawkes 2007; Perkins *et al.* 2008). Nonetheless, the accumulation of parasite species appears to be slow relative to the population growth of many introduced hosts.

Our data only address regional pathogen species richness, not impacts on hosts. The presence of enemies in the regional pool is a pre-requisite for any impacts on host populations. However, the realized impacts of natural enemies on introduced host populations may vary, depending on factors including environmental conditions, the relationship between regional and local richness of enemies, enemy species composition and each enemy’s host range (Colautti *et al.* 2004; Parker & Gilbert 2004; Torchin & Mitchell 2004; Perkins *et al.* 2008; Blumenthal *et al.* 2009). Many natural enemies of introduced species are host generalists that attack multiple sympatric host species, including natives (Mitchell *et al.* 2006; Parker *et al.* 2006; Kelly *et al.* 2009). In this case, indirect effects including apparent competition can cause enemies’ net impacts on an introduced host population to be negative or positive (Colautti *et al.* 2004; Mitchell *et al.* 2006; Perkins *et al.* 2008). Thus, while the accumulation of natural enemies by introduced species is a key component of integration into native communities, the complex structure of native communities means that this integration can have a range of ecological consequences.

Over a larger geographic range, a host may occur in more habitat types, supporting different pathogens (Nunn *et al.* 2003; Lindenfors *et al.* 2007). In models excluding habitat richness, native range pathogen richness was correlated with range size (Mitchell & Power 2003; Torchin & Mitchell 2004). In our models including habitat richness pathogen richness was correlated with habitat richness, but not range size. These results suggest that habitat richness was the chief mechanism by which range size influenced pathogen richness.

While pathogen richness was independent of host phylogeny, this does not rule out a role for host phylogeny in pathogen accumulation. The host range of many pathogens is constrained by host phylogenetic relatedness (Parker & Gilbert 2004; Gilbert & Webb 2007). Thus, introduced species that are more closely related to native species may accumulate more natural enemies (Parker & Gilbert 2004; Mitchell *et al.* 2006).

In contrast to the introduced range, host biological traits were of similar importance to historic/geographic factors in explaining native range pathogen richness. Specifically, plants adapted to abundant soil resources supported over 50% more pathogen species than those adapted to limited soil resources. The difference in importance of plant traits between native and introduced ranges does not mean that traits are not important in biological invasions. Rather, this difference explains why plants adapted to abundant soil resources experience greater absolute pathogen release (decrease in number of pathogen species) when they are introduced (Blumenthal *et al.* 2009). Our measure of resource adaptation, a single variable with two levels (stress tolerance), is a simplification of a multifaceted strategy involving ecophysiological traits, growth rate, biomass allocation, life history, palatability and other traits (Grime *et al.* 1997). Its importance in determining native range pathogen richness suggests that multiple integrated biological traits influence pathogen richness, perhaps by controlling plant susceptibility to infection (Cronin *et al.* 2010).

## SUPPORTING INFORMATION

**Appendix S1** References cited for estimation of host minimum residence times.

**Appendix S2** References cited for the construction of the phylogenetic tree.

**Figure S1** The hypothesized phylogenetic relationship of the 124 host species.

**Figure S2** Biological and historic/geographic factors explaining pathogen species richness in hosts’ native range.

**Figure S3** Bivariate plots of historic/geographic factors explaining pathogen species richness and proportional release from pathogens in hosts’ introduced range.

**Figure S4** Analysis of all data available for each response variable, rather than their intersection.

**Figure S5** Analysis of introduced range data using host habitat richness as an explanatory variable instead of residence time.

**Table S1** Parameter estimates and tests for the global model of native range pathogen richness.

**Table S2** Parameter estimates and tests for the global model of introduced range pathogen richness.

**Table S3** Parameter estimates and tests for the global model of pathogen release.

As a service to our authors and readers, this journal provides supporting information supplied by the authors. Such materials are peer-reviewed and may be re-organized for online delivery, but are not copy-edited or typeset. Technical support issues arising from supporting information (other than missing files) should be addressed to the authors.
