# Supplementary material for: Controls on pathogen species richness in plants’ introduced and native ranges: roles of residence time, range size and host traits
Source: Ecol Lett. 2010 Dec;13(12):1525–35. doi: 10.1111/j.1461-0248.2010.01543.x (PMC3003901; doi:10.1111/j.1461-0248.2010.01543.x)
Supplement: Supplementary file 1 [file ele0013-1525-SD1.doc]

**SUPPORTING INFORMATION**

The following Supporting Information is available for this article:

**Appendix S1.** References cited for estimation of host minimum residence times.

**Appendix S2.** References cited for the construction of the phylogenetic tree.

**Table S1**. Parameter estimates and tests for the global model of native range pathogen richness.

**Table S2**. Parameter estimates and tests for the global model of introduced range pathogen richness.

**Table S3**. Parameter estimates and tests for the global model of pathogen release.

**Figure S1**. The hypothesized phylogenetic relationship of the 124 host species.

**Figure S2**. Biological and historic / geographic factors explaining pathogen species richness in hosts’ native range.

**Figure S3**. Analysis of all data available for each response variable, rather than their intersection.

**Figure S4**. Analysis of introduced range data using host habitat richness as an explanatory variable instead of residence time.

Additional Supporting Information may be found in the online version of this article.

Please note: Blackwell Publishing is not responsible for the content or functionality of any supporting information supplied by the authors. Any queries (other than missing material) should be directed to the corresponding author for the article.

**Appendix S1.** References cited for estimation of host minimum residence times.

Aarssen, L. W. 1981 The biology of Canadian weeds. 50. *Hypochoeris radicata* L. *Canadian Journal of Plant Science* **61**, 365-381.

Aarssen, L. W., Hall, I. V. & Jensen, K. I. N. 1986 The biology of Canadian weeds. 76. *Vicia angustifolia* L., *Vicia cracca* L., *Vicia sativa* L., *Vicia tetrasperma* (L.) Schreb and *Vicia villosa* Roth. *Canadian Journal of Plant Science* **66**, 711-737.

Adams, D. W. 2004 *Restoring American Gardens: An Encyclopedia of Heirloom Ornamental Plants - 1640-1940* Portland, OR: Timber Press, Incorporated

Applegate, R. D. 2006 Alien Plant Invaders of Natural Areas Fact Sheets: Tall Fescue, vol. 2008. Washington D.C.: Plant Conservation Alliance Alien Plant Working Group.

Bellue, M. K., Britton, E. A. & Fuller, T. C. 1959 *Weed seed handbook : descriptions and illustrations of the primary and secondary noxious-weeds as included in California Seed Law*. Sacramento CA: California Department of Agriculture (Special Publication Number 275).

Betts, E. M., Perkins, H. B. & Hatch, P. J. 1986 *Thomas Jefferson's flower garden at Monticello* Charlottesville, VA: University Press of Virginia.

Britton, N. L. & Brown, A. 1898 *An illustrated flora of the northern United States, Canada and the British possessions from Newfoundland to the parallel of the southern boundary of Virginia, and from the Atlantic ocean westward to the 102d meridian, Vol. III*. New York: Charles Scribner's Sons.

Brooks, M. L. 2000 *Bromus madritensis* ssp. *rubens* (L.) Husnot. In *Invasive Plants of California's Wildlands* (ed. C. C. Bossard, J. M. Randall & M. C. Hoshovsky), pp. 72-76. Berkeley, CA: University of California Press.

Carrier, L. 1923 *The Beginnings of Agriculture in America*. New York: McGraw-Hill.

Crockett, L. J. 1977 *Wildly successful plants : A Handbook of North American Weeds* New York City, NY: Macmillan.

de Schweinitz, L. D. 1832 Remarks on the plants of Europe which have become naturalized in a more ore less degree, in the United States. *Annals of the Lyceum of Natural History of New York* **3**, 148-155.

Desrochers, A. M., Bain, J. F. & Warwick, S. I. 1988 The biology of Canadian weeds. 89. Carduus nutans L. and Carduus acanthoides L. *Canadian Journal of Plant Science* **68**, 1053-1068.

Gray, A. 1848 *A manual of the botany of the northern United States, from New England to Wisconsin and south to Ohio and Pennsylvania inclusive, (the mosses and liverworts by Wm. S. Sullivant), arranged according to the natural system*. Boston, MA: James Monroe and Company.

Harper, R. M. 1944 *Preliminary Report on the Weeds of Alabama*. Bulletin (Geological Survey of Alabama). Wetumpka, AL: Wetumpka Print Company.

Harshberger, J. W. 1899 *The botanists of Philadelphia and their work*. Philadelphia, PA: T. C. Davis & Son.

Haughton, C. S. 1980 *Green Immigrants: The Plants That Transformed America* New York City, NY: Harcourt, Brace, Jovanovich.

Hedrick, U. 1950 *A History of Horticulture in America to 1860*. New York: Oxford University Press.

Hoban, G. & Hoshovsky, M. C. 2000 *Erechtites glomerata / E. minima*. In *Invasive Plants of California's Wildlands* (ed. C. C. Bossard, J. M. Randall & M. C. Hoshovsky), pp. 179-183. Berkeley, CA: University of California Press.

Holm, L., Doll, J., Holm, E., Pancho, J. V. & Herberger, J. P. 1997 *World Weeds: Natural Histories and Distribution* Indianapolis, IN Wiley Publishing, Inc.

Jefferson, T. 1944 *Thomas Jefferson's Garden book, 1766-1824, with relevant extracts from his other writings, annotated by Edwin Morris Betts*. Memoirs of the American Philosophical Society Philadelphia, PA: The American Philosophical Society.

Jepson, W. L. 1925 *A Manual of the Flowering Plants of California*. Berkeley CA: University of California.

Kalm, P. 1937 *The America of 1750; Peter Kalm's Travels in North America; The English Version of 1770, revised from the original Swedish and edited by Adolph B. Benson ...* New York: Wilson-Erickson Inc.

Kline, N. 1996 *Viburnum opulus* var. *opulus*: Guelder Rose. In *Invasive plants: Weeds of the Global Garden* (ed. J. M. Randall & J. Marinelli), pp. 71. Brooklyn, NY Brooklyn Botanic Garden.

Leighton, A. 1986a *American Gardens in the Eighteenth Century: "For Use or for Delight"*. Amherst, MA: University of Massachusetts Press

Leighton, A. 1986b *Early American Gardens: "For Meate or Medicine"*. Amherst, MA: University of Massachusetts Press

Leighton, A. 1987 *American Gardens of the Nineteenth Century: "For Comfort and Affluence"*. Amherst, MA: University of Massachusetts Press

Lemna, W. K. & Messersmith, C. G. 1990 The biology of Canadian weeds. 94. Sonchus arvensis L. *Canadian Journal of Plant Science* **70**, 509-532.

Lippincott, C. & McDonald, S. 1996 *Imperata cylindrica*: Cogongrass. In *Invasive plants: Weeds of the Global Garden* (ed. J. M. Randall & J. Marinelli), pp. 88. Brooklyn, NY Brooklyn Botanic Garden.

Luken, J. O. 1996 *Lonicera maackii, L. morrowii, L. tatarica*: Bush honeysuckle. In *Invasive plants: Weeds of the Global Garden* (ed. J. M. Randall & J. Marinelli), pp. 60-61. Brooklyn, NY Brooklyn Botanic Garden.

Mack, R. N. 1991 The commercial seed trade: an early disperser of weeds in the United States. *Economic Botany* **45**, 257-273.

McNeill, J. 1980 The biology of Canadian weeds. 46. Silene noctiflora L. *Canadian Journal of Plant Science* **60**, 1243-1253.

Mehrhoff, L., Silander Jr., J., Leicht, S. A., Mosher, E. & Tabak, N. 2003 IPANE: Invasive Plant Atlas of New England, vol. 2008. Storrs, CT: Department of Ecology & Evolutionary Biology, University of Connecticut.

Mulligan, G. A. & Bailey, L. G. 1975 Biology of Canadian weeds. 8. Sinapis arvensis L. *Canadian Journal of Plant Science* **55**, 171-183.

Remaley, T. & Swearingen, J. M. 2006 Alien Plant Invaders of Natural Areas Fact Sheets: White Poplar, vol. 2008. Washington D.C.: Plant Conservation Alliance Alien Plant Working Group.

Robbins, W., Bellue, M. & Ball, W. 1951 *Weeds of California*. Sacramento, CA: California Deptartment of Agriculture.

Rousseau, C. 1968 Histoire, habitat et distribution de 220 plantes introduites au Quebec. *Le Naturaliste Canadien* **95**, 49-169.

Royer, F. & Dickinson, R. 1999 *Weeds of the Northern U.S. and Canada: A Guide for Identification*. Alberta: The University of Alberta Press

Smiley, F. J. 1922 *Weeds of California and methods of control*. Sacramento CA: California State Printing Office.

Thunhorst, G. & Swearingen, J. M. 2006 Alien Plant Invaders of Natural Areas Fact Sheets: Leafy Spurge, vol. 2008. Washington D.C.: Plant Conservation Alliance Alien Plant Working Group.

Turkington, R. & Franko, G. D. 1980 The biology of Canadian weeds. 41. *Lotus corniculatus* L. *Canadian Journal of Plant Science* **60**, 965-979.

Warwick, S. I. 1979 Biology of Canadian weeds. 37. Poa annua L. *Canadian Journal of Plant Science* **59**, 1053-1066.

Wiesenborn, W. 1996 *Tamarix ramosissima, T. chinensis, T. parviflora*: Tamarisk. In *Invasive plants: Weeds of the Global Garden* (ed. J. M. Randall & J. Marinelli), pp. 44-45. Brooklyn, NY Brooklyn Botanic Garden.

**Appendix S2.** References cited for the construction of the phylogenetic tree.

Bailey, C. D., Koch, M. A., Mayer, M., Mummenhoff, K., O'Kane, S. L., Warwick, S. I., Windham, M. D. & Al-Shehbaz, I. A. 2006 Toward a global phylogeny of the Brassicaceae. *Molecular Biology and Evolution* **23**, 2142-2160.

Beilstein, M. A., Al-Shehbaz, I. A., Mathews, S. & Kellogg, E. A. 2008 Brassicaceae phylogeny inferred from phytochrome A and ndhF sequence data: tribes and trichomes revisited. *Am. J. Bot.* **95**, 1307-1327.

Bouchenak-Khelladi, Y., Salamin, N., Savolainen, V., Forest, F., van der Bank, M., Chase, M. W. & Hodkinson, T. R. 2008 Large multi-gene phylogenetic trees of the grasses (Poaceae): Progress towards complete tribal and generic level sampling. *Molecular Phylogenetics and Evolution* **47**, 488-505.

Donoghue, M. J., Baldwin, B. G., Li, J. & Winkworth, R. C. 2004 *Viburnum* Phylogeny Based on Chloroplast trnK Intron and Nuclear Ribosomal ITS DNA Sequences. *Systematic Botany* **29**, 188-198.

Downie, S. R., Katz-Downie, D. S. & Watson, M. F. 2000 A phylogeny of the flowering plant family Apiaceae based on chloroplast DNA rpl16 and rpoC1 intron sequences: towards a suprageneric classification of subfamily Apioideae. *Am. J. Botany* **87**, 273-292.

Enke, N. & Gemeinholzer, B. 2008 Babcock revisited: new insights into generic delimitation and character evolution in Crepis L. (Compositae : Cichorieae) from ITS and matK sequence data. *Taxon* **57**, 756-768.

Fior, S., Karis, P. O., Casazza, G., Minuto, L. & Sala, F. 2006 Molecular phylogeny of the Caryophyllaceae (Caryophyllales) inferred from chloroplast MATK and nuclear rDNA its sequences. *American Journal of Botany* **93**, 399-411.

Funk, V. A., Bayer, R. J., Keeley, S., Chan, R., Watson, L., Gemeinholzer, B., Schilling, E., Panero, J. L., Baldwin, B. G., Garcia-Jacas, N., Susanna, A. & Jansen, R. K. 2005 Everywhere but Antarctica: Using a supertree to understand the diversity and distribution of the Compositae. *Biologiske skrifter* **55**, 343-374.

Garcia-Jacas, N., Susanna, A., Garnatje, T. & Vilatersana, R. 2001 Generic delimitation and phylogeny of the subtribe Centaureinae (Asteraceae): A combined nuclear and chloroplast DNA analysis. *Annals of Botany* **87**, 503-515.

Johansson, J. T. & Jansen, R. K. 1993 Chloroplast DNA variation and phylogeny of the Ranunculaceae. *Plant Systematics and Evolution* **187**, 29-49.

Kim, S. C., Chunghee, L. & Mejias, J. A. 2007 Phylogenetic analysis of chloroplast DNA matK gene and ITS of nrDNA sequences reveals polyphyly of the genus Sonchus and new relationships among the subtribe Sonchinae (Asteraceae : Cichorieae). *Molecular Phylogenetics and Evolution* **44**, 578-597.

Långström, E. & Chase, M. W. 2002 Tribes of Boraginoideae (Boraginaceae) and placement of *Antiphytum* , *Echiochilon* , *Ogastemma* and *Sericostoma* : A phylogenetic analysis based on atp B plastid DNA sequence data. *Plant Systematics and Evolution* **234**, 137-153.

Li, R.-Q., Chen, Z.-D., Lu, A.-M., Soltis, D. E., Soltis, P. S. & Manos, P. S. 2004 Phylogenetic Relationships in Fagales Based on DNA Sequences from Three Genomes. *International Journal of Plant Sciences* **165**, 311-324.

Manos, Paul S., Zhou, Z.-K. & Cannon, Charles H. 2001 Systematics of Fagaceae: Phylogenetic Tests of Reproductive Trait Evolution. *International Journal of Plant Sciences* **162**, 1361-1379.

Muller, K. & Borsch, T. 2005 Phylogenetics of Amaranthaceae Based on matK/trnK Sequence Data: Evidence from Parsimony, Likelihood, and Bayesian Analyses. *Annals of the Missouri Botanical Garden* **92**, 66-102.

Oh, S.-H. & Manos, P. S. 2008 Molecular phylogenetics and cupule evolution in Fagaceae as inferred from nuclear CRABS CLAW sequences. *Taxon* **57**, 434-451.

Popp, M. & Oxelman, B. 2004 Evolution of a RNA polymerase gene family in *Silene* (Caryophyllaceae) - Incomplete concerted evolution and topological congruence among paralogues. *Systematic Biology* **53**, 914-932.

Potokina, E., Tomooka, N., Vaughan, D. A., Alexandrova, T. & Xu, R. Q. 1999 Phylogeny of *Vicia* subgenus *Vicia* (Fabaceae) based on analysis of RAPDs and RFLP of PCR-amplified chloroplast genes. *Genetic Resources and Crop Evolution* **46**, 149-161.

Potter, D., Eriksson, T., Evans, R. C., Oh, S., Smedmark, J. E. E., Morgan, D. R., Kerr, M., Robertson, K. R., Arsenault, M., Dickinson, T. A. & Campbell, C. S. 2007 Phylogeny and classification of Rosaceae. *Plant Systematics and Evolution* **266**, 5-43.

Pyck, N., Roels, P. & Smets, E. 1999 Tribal Relationships in Caprifoliaceae: Evidence from a Cladistic Analysis Using ndhF Sequences. *Systematics and Geography of Plants* **69**, 145-159.

Roquet, C., Saez, L., Aldasoro, J. J., Susanna, A., Alarcon, M. L. & Garcia-Jacas, N. 2008 Natural delineation, molecular phylogeny and floral evolution in *Campanula*. *Systematic Botany* **33**, 203-217.

Scheen, A. C., Brochmann, C., Brysting, A. K., Elven, R., Morris, A., Soltis, D. E., Soltis, P. S. & Albert, V. A. 2004 Northern hemisphere biogeography of *Cerastium* (Caryophyllaceae): Insights from phylogenetic analysis of noncoding plastid nucleotide sequences. *American Journal of Botany* **91**, 943-952.

Tank, D. C., Beardsley, P. M., Kelchner, S. A. & Olmstead, R. G. 2006 Review of the systematics of Scrophulariaceae *s.l.* and their current disposition. *Australian Systematic Botany* **19**, 289-307.

Torrecilla, P., Lopez-Rodriguez, J. A. & Catalan, P. 2004 Phylogenetic relationships of *Vulpia* and related genera (Poeae, Poaceae) based on analysis of ITS and trnL-F sequences. *Annals of the Missouri Botanical Garden* **91**, 124-158.

Wagstaff, S. J., Hickerson, L., Spangler, R., Reeves, P. A. & Olmstead, R. G. 1998 Phylogeny in Labiatae sl, inferred from cpDNA sequences. *Plant Systematics and Evolution* **209**, 265-274.

Walker, J. B., Sytsma, K. J., Treutlein, J. & Wink, M. 2004 *Salvia* (Lamiaceae) is not monophyletic: implications for the systematics, radiation, and ecological specializations of *Salvia* and tribe Mentheae. *American Journal of Botany* **91**, 1115-1125.

Warwick, S. I. & Sauder, C. A. 2005 Phylogeny of tribe Brassiceae (Brassicaceae) based on chloroplast restriction site polymorphisms and nuclear ribosomal internal transcribed spacer and chloroplast trnL intron sequences. *Canadian Journal of Botany-Revue Canadienne De Botanique* **83**, 467-483.

Table S1. Parameter estimates (with asymptotic standard errors) and Type III quasi-likelihood Wald tests for the global model of native range pathogen richness. Shown χ^2^ values were scaled by the model’s residual deviance divided by its degrees of freedom.

| **Parameter** | **Estimate ± Std Error** | **DF** | **χ^2^** | **p-value** |
| --- | --- | --- | --- | --- |
| Intercept | 1.236 ± 0.4228 | 1 | 8.55 | 0.0035 |
| Sampling effort [ln(native range cites)] | 0.1206 ± 0.0398 | 1 | 9.16 | 0.0025 |
| Stress tolerance (yes) | -0.3317 ± 0.1274 | 1 | 6.78 | 0.0092 |
| Height [ln(m)] | -0.000177 ± 0.000512 | 1 | 0.119 | 0.73 |
| Leaf type (thickened epidermis and cuticle) | 0.2303 ± 0.1247 | 1 | 3.41 | 0.065 |
| History of agricultural use (yes) | 0.3085 ± 0.1291 | 1 | 5.71 | 0.017 |
| Native geographic range size (10^6^ km^2^) | 0.0214 ± 0.0324 | 1 | 0.437 | 0.51 |
| Habitat richness (number of types) | 0.0169 ± 0.0040 | 1 | 17.6 | <0.0001 |

Table S2. Parameter estimates (with asymptotic standard errors) and Type III quasi-likelihood Wald tests for the global model of introduced range pathogen richness. Shown χ^2^ values were scaled by the model’s residual deviance divided by its degrees of freedom.

| **Parameter** | **Estimate ± Std Error** | **DF** | **χ^2^** | **p-value** |
| --- | --- | --- | --- | --- |
| Intercept | -1.255 ± 0.6478 | 1 | 3.76 | 0.053 |
| Sampling effort [ln(introduced range cites)] | 0.1238 ± 0.0600 | 1 | 4.25 | 0.039 |
| Stress tolerance (yes) | -0.5192 ± 0.3149 | 1 | 2.72 | 0.099 |
| Height [ln(m)] | -0.000818 ± 0.000857 | 1 | 0.911 | 0.34 |
| Leaf type (thickened epidermis and cuticle) | 0.2985 ± 0.2280 | 1 | 1.71 | 0.19 |
| History of agricultural use (yes) | 1.263 ± 0.2407 | 1 | 27.5 | <0.0001 |
| Introduced geographic range size (10^6^ km^2^) | 0.2333 ± 0.0515 | 1 | 20.5 | <0.0001 |
| Residence time (centuries) | 0.5240 ± 0.1197 | 1 | 19.1 | <0.0001 |

Table S3. Parameter estimates (with asymptotic standard errors) and Type III quasi-likelihood Wald tests for the global model of introduced range proportional pathogen release. Shown χ^2^ values were scaled by the model’s residual deviance divided by its degrees of freedom.

| **Parameter** | **Estimate ± Std Error** | **DF** | **χ^2^** | **p-value** |
| --- | --- | --- | --- | --- |
| Intercept | 2.801 ± 0.9339 | 1 | 9.00 | 0.0027 |
| Sampling effort [ln(native)-ln(introduced)] | 0.1901 ± 0.1135 | 1 | 2.80 | 0.094 |
| Stress tolerance (yes) | -0.4114 ± 0.4124 | 1 | 0.995 | 0.32 |
| Height [ln(m)] | 0.000991 ± 0.001317 | 1 | 0.567 | 0.45 |
| Leaf type (thickened epidermis and cuticle) | -0.2720 ± 0.3261 | 1 | 0.700 | 0.40 |
| History of agricultural use (yes) | -1.166 ± 0.3052 | 1 | 14.6 | 0.0001 |
| Introduced geographic range size (10^6^ km^2^) | -0.2649 ± 0.0713 | 1 | 13.8 | 0.0002 |
| Residence time (centuries) | -0.6234 ± 0.1680 | 1 | 13.8 | 0.0002 |

Figure S1. The hypothesized phylogenetic relationship of the 124 host species. The scale bar unit is millions of years ago.

Figure S2. Biological and historic / geographic factors explaining pathogen species richness in hosts’ native range. Gray symbols indicate stress tolerant hosts, and black symbols indicate hosts that are not stress tolerant. Diamonds indicate hosts with a history of agricultural use, and circles indicate hosts with no history of agricultural use. Symbols with cross-hairs indicate hosts with a thickened leaf cuticle and epidermis, and symbols without cross-hairs indicate hosts without this leaf anatomy. Shown χ^2^ values are from quasi-likelihood Wald tests and were scaled by the model’s residual deviance divided by its degrees of freedom. Statistics are from the AIC best model, which included only sampling effort (log‐transformed citation count) and the five explanatory variables shown. Results were similar in all models analyzed. Pathogen species richness was greater on hosts that occupied a greater number of habitat types (χ^2^_1_ = 18.0; p<0.0001), on hosts that were not stress tolerant (χ^2^_1_ = 6.74; p=0.0094), and on hosts with a history of agricultural use (χ^2^_1_ = 5.65; p=0.017). Pathogen species richness also tended to be greater on hosts with a thickened leaf cuticle and epidermis (χ^2^_1_ = 3.44; p=0.064), and on hosts with a larger native geographic range size (χ^2^_1_ = 0.65; p=0.42).

Figure S3. Historic / geographic factors explaining pathogen species richness and proportional release from pathogens in hosts’ introduced range. (A) Pathogen richness was greater on hosts that had a larger introduced geographic range size (χ^2^_1_ = 45.5; p<0.0001). (B) Pathogen richness was greater on hosts that had a longer residence time in the introduced range (χ^2^_1_ = 9.99; p<0.0016). (C) Release from pathogens was lesser on hosts that had a larger introduced geographic range size (χ^2^_1_ = 40.2; p<0.0001). (D) Release from pathogens was lesser on hosts that had a longer residence time in the introduced range (χ^2^_1_ = 5.11; p=0.024). (A-D) When points had identical x and y coordinates, the x-coordinate was jittered to render all points visible.

Figure S4. The relative importance (on a scale from 0 to 1) of biological (black bars) and historic / geographic (gray bars) variables in explaining (A) pathogen richness in hosts’ native ranges (n=218), (B) pathogen richness in hosts’ introduced ranges (n=175), (C) pathogen release in hosts’ introduced ranges (n=158). These results use all available data for each of the three response variables. Results were similar to those in Fig. 1, which were based on the intersection of these three expanded data sets.

Figure S5. The relative importance (on a scale from 0 to 1) of biological (black bars) and historic / geographic (gray bars) variables in explaining (A) pathogen richness in hosts’ introduced ranges, and (B) pathogen release in hosts’ introduced ranges. Host habitat richness was used as an explanatory variable instead of residence time. Results were similar to the analogous main analyses (Fig. 1.B,C).

Figure S1.


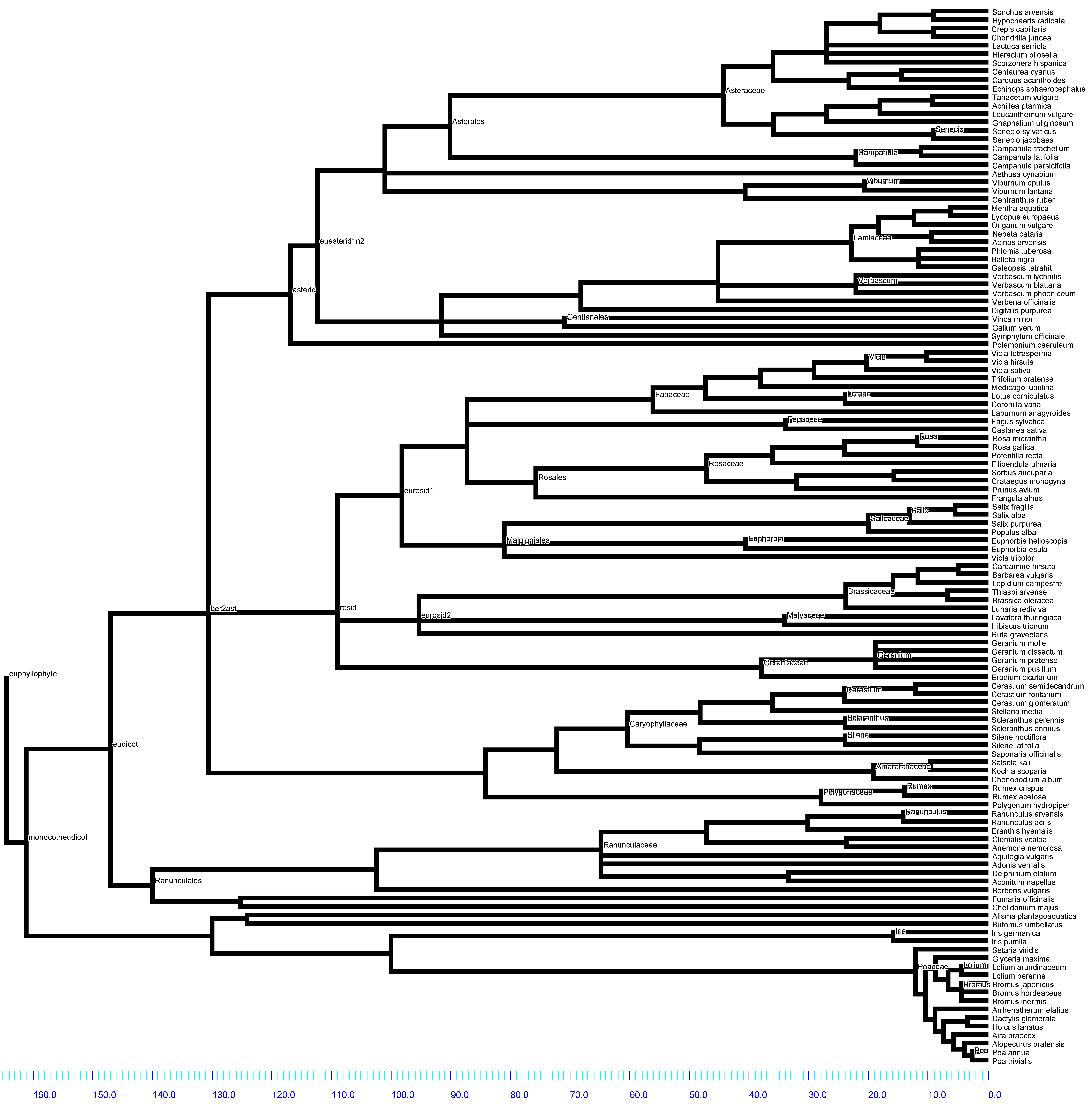


Figure S2.

Figure S3.

Figure S4.

Figure S5.
